# Single-cell RNA sequencing to decipher the immunogenicity of ChAdOx1 nCoV-19/AZD1222 and mRNA-1273 vaccines in patients with autoimmune rheumatic diseases

**DOI:** 10.3389/fimmu.2022.920865

**Published:** 2022-08-01

**Authors:** Yen-Ju Chen, Po-Liang Cheng, Wen-Nan Huang, Hsin-Hua Chen, Hong-Wei Chen, Jun-Peng Chen, Ching-Tsai Lin, Kuo-Tung Tang, Wei-Ting Hung, Tsu-Yi Hsieh, Yi-Hsing Chen, Yi-Ming Chen, Tzu-Hung Hsiao

**Affiliations:** ^1^ Division of Allergy, Immunology, and Rheumatology, Department of Internal Medicine, Taichung Veterans General Hospital, Taichung, Taiwan; ^2^ Institute of Clinical Medicine, National Yang-Ming Chiao Tung University, Taipei, Taiwan; ^3^ Department of Medical Research, Taichung Veterans General Hospital, Taichung, Taiwan; ^4^ School of Medicine, National Yang-Ming Chiao Tung University, Taipei, Taiwan; ^5^ Department of Pathology and Laboratory Medicine, Perelman School of Medicine, University of Pennsylvania, Philadelphia, PA, United States; ^6^ Department of Post-Baccalaureate Medicine, College of Medicine, National Chung Hsing University, Taichung, Taiwan; ^7^ Department of Industrial Engineering and Enterprise Information, Tunghai University, Taichung, Taiwan; ^8^ Institute of Biomedical Science and Rong Hsing Research Center for Translational Medicine, National Chung Hsing University, Taichung, Taiwan; ^9^ Institute of Public Health and Community Medicine Research Center, National Yang-Ming University, Taipei, Taiwan; ^10^ Department of Medical Education, Taichung Veterans General Hospital, Taichung, Taiwan; ^11^ Department of Public Health, Fu Jen Catholic University, New Taipei City, Taiwan; ^12^ Institute of Genomics and Bioinformatics, National Chung Hsing University, Taichung, Taiwan

**Keywords:** COVID-19 vaccine, rheumatic disease, autoimmune, anti-rheumatic medications, single-cell RNA sequencing

## Abstract

**Objectives:**

To investigate the differences between the vector vaccine ChAdOx1 nCoV-19/AZD1222 (Oxford-AstraZeneca) and mRNA-based vaccine mRNA-1273 (Moderna) in patients with autoimmune rheumatic diseases (AIRD), and to explore the cell-cell interactions between high and low anti-SARS-CoV-2 IgG levels in patients with rheumatic arthritis (RA) using single-cell RNA sequencing (scRNA-seq).

**Methods:**

From September 16 to December 10, 2021, we consecutively enrolled 445 participants (389 patients with AIRD and 56 healthy controls), of whom 236 were immunized with AZD1222 and 209 with mRNA-1273. The serum IgG antibodies to the SARS-CoV-2 receptor-binding domain was quantified by electrochemiluminescence immunoassay at 4-6 weeks after vaccination. Moreover, peripheral blood mononuclear cells (PBMCs) were isolated from RA patients at 4-6 weeks after vaccination for scRNA-seq and further analyzed by CellChat. ScRNA-seq of PBMCs samples from GSE201534 in the Gene Expression Omnibus (GEO) database were also extracted for analysis.

**Results:**

The anti-SARS-CoV-2 IgG seropositivity rate was 85.34% for AIRD patients and 98.20% for healthy controls. The anti-SARS-CoV-2 IgG level was higher in patients receiving mRNA-1273 than those receiving AZD1222 (β: 35.25, 95% CI: 14.81-55.68, p=0.001). Prednisolone-equivalent dose >5 mg/day and methotrexate use in AIRD patients, and non-anti-tumor necrosis factor-α biologics and Janus kinase inhibitor use in RA patients were associated with inferior immunogenicity. ScRNA-seq revealed CD16^-^monocytes were predominant in RA patients with high anti-SARS-CoV2-IgG antibodies, and enriched pathways related to antigen presentation *via* MHC class II were found. HLA-DRA and CD4 interaction was enhanced in high anti-SARS-CoV2-IgG group.

**Conclusions:**

mRNA-1273 and AZD1222 vaccines exhibited differential immunogenicity in AIRD patients. Enriched pathways related to antigen presentation *via* MHC class II in CD16^-^monocytes might be associated with higher anti-SARS-CoV2-IgG level in RA patients and further study is warranted.

## Introduction

Worldwide, the coronavirus disease (COVID-19) pandemic remains a threat to public health and socioeconomic conditions. Autoimmune rheumatic diseases (AIRD) are chronic systemic inflammatory diseases that disrupt immune responses in approximately 4% of the global population (1, 2). Patients with rheumatic diseases are vulnerable to severe acute respiratory distress syndrome coronavirus 2 (SARS-CoV-2) infection (3, 4). However, not all immunosuppressed patients experience serious illness following SARS-CoV-2 infection, and this may be partly attributed to the differences in the immune disorders and the use of different immunosuppressive agents (5, 6).

The prevention of COVID-19 is a top priority and is vital for restoring normalcy. Previous studies have demonstrated COVID-19 vaccines, mostly mRNA-based vaccines, could elicit adequate immunogenicity in AIRD patients (7, 8). Although a lower percentage of rheumatic disease patients achieved seropositivity and had a relatively lower anti-SARS-CoV-2 immunoglobulin G (IgG) level, compared with healthy controls, these patients did not experience a flaring up of rheumatic diseases nor an increased number of adverse reactions with COVID-19 vaccines (8). The reduced immunogenicity in AIRD patients was probably attributable to the immune disturbance due to rheumatic disease or the immunosuppressive effects of concomitant glucocorticoids, conventional synthetic disease-modifying antirheumatic drugs (csDMARDs), biological DMARDs (bDMARDs), or targeted-synthetic DMARDs (tsDMARDs). Haberman et al. demonstrated that a smaller proportion of rheumatic patients on methotrexate (MTX) achieved adequate immunogenicity compared with non-users (9). Furer et al. showed that patients using glucocorticoids, mycophenolate mofetil (MMF), and non-tumor necrosis factor (TNF)-α bDMARDs, mainly abatacept and rituximab, achieved lower immunogenicity after receiving the BNT162b2 mRNA vaccine (8).

Most of the previous studies focused on the newly developed mRNA-based vaccines. However, there are fewer reports of the immunogenicity of vector vaccines, such as AZD1222, in AIRD patients. Furthermore, differences between mRNA-based and vector vaccines with regard to the immune response and safety profile of these vaccines in patients with rheumatic disease are unclear. Besides, there is a lack of information about the impact of immunosuppressants on immunogenicity in patients with rheumatic disease, especially those who were receiving tsDMARDs. In addition, no previous reports made a thorough exploration of the cellular responses to COVID-19 vaccines according to different immunogenicity in AIRD patients.

Therefore, we aimed to explore the differences in efficacy and safety between the vector vaccine AZD1222 and the mRNA-based vaccine mRNA-1273 as well as the impact of anti-rheumatic medications on vaccine-induced immunogenicity in AIRD patients, and to investigate the cell-cell interactions between high and low anti-SARS-CoV-2 IgG levels in patients with rheumatoid arthritis (RA) by single-cell RNA sequencing (scRNA-seq). The primary objective of this study was to elucidate the immune response induced by COVID-19 vaccination with either AZD1222 (Oxford-AstraZeneca) or mRNA-1273 (Moderna) in a cohort of patients with rheumatic diseases in a tertiary medical center in Taiwan.

## Materials and methods

### Study participants

From September 16 to December 10, 2021, we consecutively enrolled adult patients (age ≥20 years) with rheumatic diseases and healthy controls who received COVID-19 vaccination at a medical center in Taichung, Taiwan. Rheumatic diseases were diagnosed according to the corresponding classification criteria and confirmed by two experienced rheumatologists (10–16). Patients with a history of SARS-CoV-2 infection were excluded. The date of COVID-19 vaccination was defined as the index date in this study.

This study was conducted in accordance with the Declaration of Helsinki and was approved (CF21297B) by the Institutional Review Board of Taichung Veterans General Hospital, Taiwan. All participants provided written informed consent prior to their enrolment in the study.

### Definition of covariates

Demographics and comorbidities were recorded besides the rheumatic disease types and anti-rheumatic medications, which comprised glucocorticoids, csDMARDs, bDMARDs and tsDMARDs such as the Janus kinase (JAK) inhibitors tofacitinib and baricitinib. Drug exposure was defined as treatment received in the 3 months preceding the index date.

The date of vaccination and type of vaccine were documented, and questionnaires were used for the surveillance of vaccine-associated adverse reactions. Moreover, the patient self-reported awareness of disease activities of underlying rheumatic diseases and physician global assessments at 4–6 weeks after the COVID-19 vaccination was recorded.

### Anti-SARS-CoV2-IgG detection

At 4–6 weeks after the first or second dose of COVID-19 vaccines, the level of serum IgG antibodies to the SARS-CoV-2 receptor-binding domain (RBD) on spike protein S1 subunit was quantified by electrochemiluminescence immunoassay in accordance with the manufacturer’s instructions (Elecsys Anti-SARS-CoV-2 S assay, Roche Diagnostics, Basel, Switzerland). The interval of analytical measurement was 0.40–250 U/mL; a value <0.8 and ≥0.80 U/mL was recorded as negative and positive, respectively. High immunogenicity following vaccination was defined as anti-SARS-COV2-IgG after vaccination equaled to 250 U/ml; low immunogenicity was defined as anti-SARS-COV2-IgG after vaccination < 10 U/ml.

### scRNA-seq datasets of healthy individuals following COVID-19 vaccination

scRNA-seq data of peripheral blood mononuclear cells (PBMCs) from 6 healthy individuals who had received 2 doses of ChAdOx1 (N=2), 2 doses of BNT162b2 (N=2), and heterologous ChAdOx1 and BNT162b2 vaccination were extracted from the National Center for Biotechnology Information (NCBI) in the Gene Expression Omnibus (GEO) Database (GSE201534). The dataset was used for cell proportions and pathway analysis.

### Cell preparation and cryopreservation

PBMCs were isolated from RA patients at 4-6 weeks after the first immunization either with AZD1222 or mRNA-1273 in EDTA tubes and isolated by Lymphoprep (STEMCELL, Vancouver, BC, Canada). Briefly, samples were diluted with phosphate-buffered saline (PBS) and layered over Ficoll-Plaque and centrifuged at 800 x *g* for 20 minutes at room temperature. PBMCs were collected, diluted with PBS and centrifuged again at 300 x *g* for 15 minutes at room temperature. After one time wash with cold PBS, Cell Freezing Medium (ScienCell, Carlsbad, CA, USA) was used to resuspend cells and transfer to cryogenic storage vials (1x 10^7^ - 1x10^8^ cells/ml). The vials were placed into the Cell Freezing Containers and stored at -80°C overnight. Vials were then stored in liquid nitrogen until use.

### Cell thawing

Within the same day before scRNA-seq experiment, frozen cells were removed from liquid nitrogen and thawed slowly in 37°C water bath for 3 minutes. Cells were then transferred to a pre-warmed 15ml falcon with 9ml Roswell Park Memorial Institute (RPMI) and 10% fetal bovine serum (FBS). Samples were incubated at room temperature for 10 minutes and centrifuged at 400 x g for 5 minutes at room temperature. Supernatant was removed and resuspended with 1ml PBS + 2% FBS gently. Cell concentration and viability were determined, and cell concentration was adjusted to approximately 1,000 cells per ml.

### ScRNA-seq library preparation and sequencing

The PBMC samples were labeled with CellMultiplex oligos and were mixed following by loading on 10x Genomics Chromium Single Cell Instrument (10x Genomics, Pleasanton, CA, USA). Chromium Next GEM Single Cell 3’Reagent Kits v3.1 (Dual Index) was used for scRNA-seq library preparation according to the manufacturer’s instructions. Gene expression and CellMultiplex libraries was loaded at a ratio of 4:1 and 300pm onto the Illumina NovaSeq 6000 with paired end kits.

### ScRNA-seq data analysis

The Fastq files were input into Cell Ranger Suite 6.0.1 for raw reads alignment, demultiplexing of barcodes, and quantification. Cells were filtered if unique feature counts > 9000 or < 200 and were eliminated as noise signals if mitochondrial counts > 10% by Seurat R package (17). SCTransform was used to normalize the post-filter matrix in order to eliminate the batch effect (18). Principal component analysis was used for dimensional reduction, and the k-nearest neighbor (KNN) graph and Louvain algorithm was performed for clustering. CellMarker database and scmap R package were used to annotate every cluster classified (19, 20). CellChat was used to investigate the cell-cell communication signal (21).

### Statistical analysis

The chi-square or Mann–Whitney *U* test was conducted to compare the efficacy and safety between AZD1222 and mRNA-1273. Linear regression analyses were used to examine the immunogenicity and the impact of anti-rheumatic medications on immunogenicity in AIRD patients, expressed as regression coefficient (β) and 95% confidence intervals (95% CI). The Kruskal–Wallis test, followed by the Dunn–Bonferroni test for *post hoc* analysis, was used to determine immunogenicity in RA patients with or without bDMARDs and tsDMARDs. The Statistical Package for the Social Sciences (SPSS) version 22.0 was used for statistical analysis. Statistical significance was indicated by *p*<0.05.

## Results

### Demographics of participants who received COVID-19 vaccination

**Figure 1:**
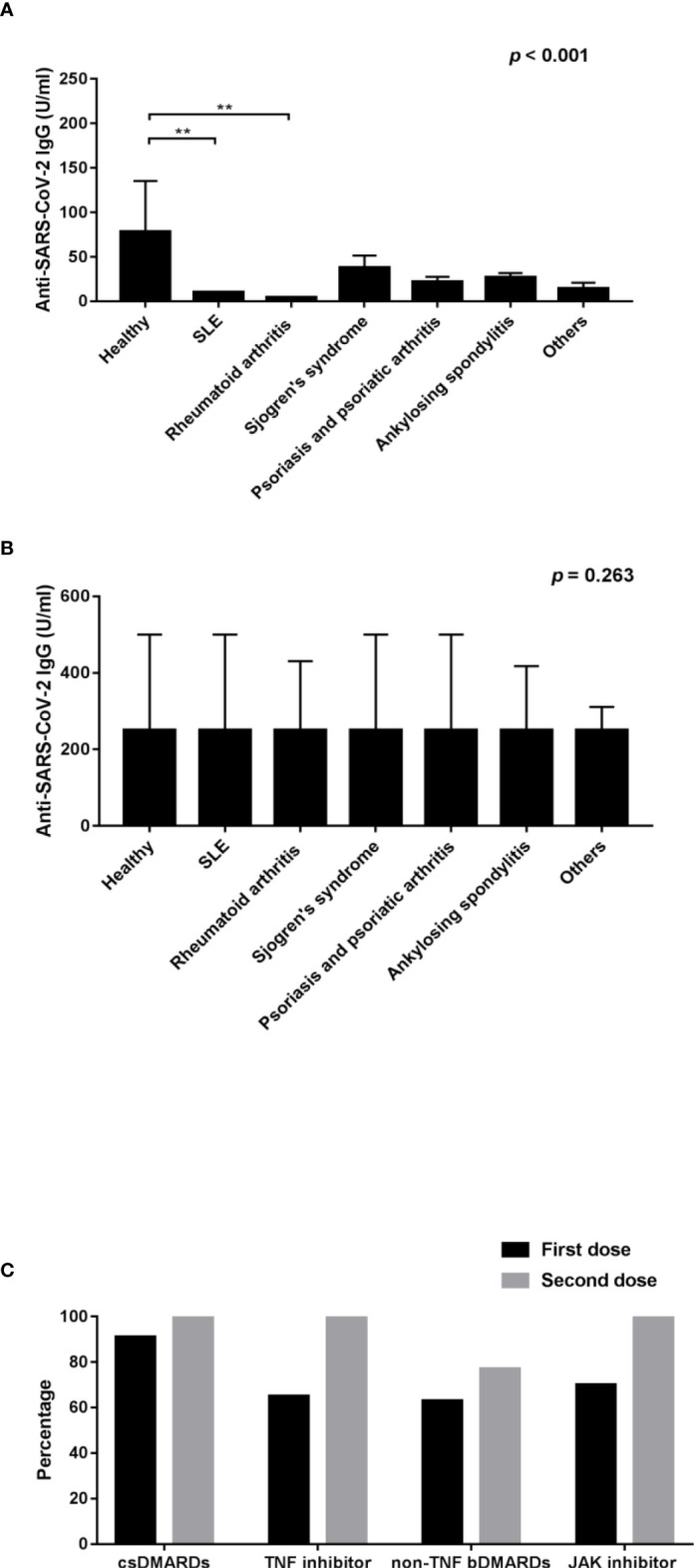
Comparison of the anti-SARS-CoV-2 IgG level after **(A)** the first dose of COVID-19 vaccines and **(B)** the second dose of COVID-19 vaccines among healthy controls and patients with individual rheumatic diseases; and **(C)** comparison of the percentage of seropositivity rate of RA patients using csDMARDs, TNF inhibitors, non-TNF bDMARDs, and JAK inhibitors after the first and second doses of COVID-19 vaccines. SLE, systemic lupus erythematosus; RA, rheumatoid arthritis; csDMARDs, conventional synthetic disease-modifying antirheumatic drugs; TNF, tumor necrosis factor; bDMARDs, biologic disease-modifying antirheumatic drugs; JAK, Janus kinase. **p < 0.001.

This study enrolled 445 patients who received COVID-19 vaccination, of whom there were 389 AIRD patients and 56 healthy controls. 236 patients were immunized with AZD1222 and 209 patients were immunized with mRNA-1273. Following vaccination, the anti-SARS-CoV-2 IgG seropositivity rate was 85.34% (332/389) and 98.2% (55/56) in AIRD patients and in healthy controls, respectively. In patients with rheumatic diseases, the seropositivity rate of anti-SARS-CoV-2 IgG was higher after the second doses of COVID-19 vaccine than after the first dose (95.2% vs. 79.6%, *p*=0.025) (**Table 1**). Among AIRD patients after the first dose of COVID-19 vaccination, there was markedly lower anti-SARS-CoV-2 IgG level in patients with SLE and RA than in healthy controls (**Figure 1A**). Nevertheless, there were no significant differences of anti-SARS-CoV-2 IgG level in patients with AIRD than in healthy controls after the second dose of COVID-19 vaccination (**Figure 1B**).

**Table 1:** Demographics and comorbidities of patients with rheumatic diseases following the first dose of COVID-19 vaccines.

Patient characteristics	Total		ChAdOx1 nCoV-19/AZD1222 (n=236)					Total		mRNA-1273 (n=209)					*p* value
			Seropositivity		No seropositivity		*p* value			Seropositivity		No seropositivity		*p* value	
			(n=207, 87.7%)		(n=29, 12.3%)					(n=180, 86.1%)		(n=29, 13.9%)			
Age, years	48.2	(38.0-57.3)	46.7	(35.6-56.4)	57.6	(46.5-62.6)	0.001	65.5	(54.6-69.8)	63.7	(54.3-69.6)	68.8	(58.2-70.7)	0.065	<0.001
Sex							0.207							0.229	0.226
Female	151	(64.0%)	136	(65.7%)	15	(51.7%)		146	(69.9%)	129	(71.7%)	17	(58.6%)		
Male	85	(36.0%)	71	(34.3%)	14	(48.3%)		63	(30.1%)	51	(28.3%)	12	(41.4%)		
Diagnosis							0.157							0.326	<0.001
Healthy controls	46	(19.5%)	45	(21.7%)	1	(3.4%)		10	(4.8%)	10	(5.6%)	0	(0.0%)		
SLE	39	(16.5%)	35	(16.9%)	4	(13.8%)		43	(20.6%)	38	(21.1%)	5	(17.2%)		
Rheumatoid arthritis	82	(34.7%)	66	(31.9%)	16	(55.2%)		112	(53.6%)	91	(50.6%)	21	(72.4%)		
Sjogren’s syndrome	12	(5.1%)	11	(5.3%)	1	(3.4%)		17	(8.1%)	15	(8.3%)	2	(6.9%)		
Psoriasis and psoriatic arthritis	17	(7.2%)	14	(6.8%)	3	(10.3%)		6	(2.9%)	6	(3.3%)	0	(0.0%)		
Ankylosing spondylitis	19	(8.1%)	17	(8.2%)	2	(6.9%)		11	(5.3%)	11	(6.1%)	0	(0.0%)		
Others	21	(8.9%)	19	(9.2%)	2	(6.9%)		10	(4.8%)	9	(5.0%)	1	(3.4%)		
Doses of COVID-19 vaccination							<0.001							0.026	0.432
The first dose	120	(50.8%)	94	(45.4%)	26	(89.7%)		115	(55.0%)	93	(51.7%)	22	(75.9%)		
The second dose	116	(49.2%)	113	(54.6%)	3	(10.3%)		94	(45.0%)	87	(48.3%)	7	(24.1%)		
Comorbidities															
Hypertension	19	(8.1%)	14	(6.8%)	5	(17.2%)	0.066	40	(19.1%)	33	(18.3%)	7	(24.1%)	0.629	0.002
Hyperlipidemia	12	(5.1%)	8	(3.9%)	4	(13.8%)	0.045	26	(12.4%)	25	(13.9%)	1	(3.4%)	0.139	0.009
CKD	9	(3.8%)	8	(3.9%)	1	(3.4%)	1.000	26	(12.4%)	21	(11.7%)	5	(17.2%)	0.373	0.001
CAD	5	(2.1%)	5	(2.4%)	0	(0.0%)	1.000	18	(8.6%)	15	(8.3%)	3	(10.3%)	0.721	0.004
CVA	4	(1.7%)	3	(1.4%)	1	(3.4%)	0.410	14	(6.7%)	12	(6.7%)	2	(6.9%)	1.000	0.015
Asthma	6	(2.5%)	6	(2.9%)	0	(0.0%)	1.000	6	(2.9%)	6	(3.3%)	0	(0.0%)	1.000	1.000
COPD	1	(0.4%)	1	(0.5%)	0	(0.0%)	1.000	6	(2.9%)	4	(2.2%)	2	(6.9%)	0.196	0.055
DM	10	(4.2%)	7	(3.4%)	3	(10.3%)	0.111	17	(8.1%)	15	(8.3%)	2	(6.9%)	1.000	0.129
Malignancy	8	(3.4%)	6	(2.9%)	2	(6.9%)	0.256	9	(4.3%)	7	(3.9%)	2	(6.9%)	0.362	0.798
Medications															
Glucocorticoid (mg/day)	0.0	(0.0-5.0)	0.0	(0.0-5.0)	2.5	(0.0-5.0)	0.220	1.4	(0.0-5.0)	0.0	(0.0-5.0)	5.0	(1.4-8.8)	0.001	0.640
Methotrexate	70	(29.7%)	57	(27.5%)	13	(44.8%)	0.091	76	(36.4%)	62	(34.4%)	14	(48.3%)	0.219	0.161
Leflunomide	16	(6.8%)	12	(5.8%)	4	(13.8%)	0.117	11	(5.3%)	8	(4.5%)	3	(10.3%)	0.185	0.648
Sulfasalazine	46	(19.5%)	40	(19.3%)	6	(20.7%)	1.000	57	(27.3%)	45	(25.0%)	12	(41.4%)	0.107	0.067
Hydroxychloroquine	105	(44.5%)	90	(43.5%)	15	(51.7%)	0.524	128	(61.2%)	109	(60.6%)	19	(65.5%)	0.761	0.001
Azathioprine	21	(8.9%)	18	(8.7%)	3	(10.3%)	0.729	23	(11.1%)	22	(12.3%)	1	(3.4%)	0.212	0.548
Mycophenolate mofetil/acid	11	(4.7%)	9	(4.3%)	2	(6.9%)	0.630	7	(3.4%)	5	(2.8%)	2	(6.9%)	0.253	0.653
Cyclosporine	12	(5.1%)	10	(4.8%)	2	(6.9%)	0.646	12	(5.8%)	10	(5.6%)	2	(6.9%)	0.676	0.914
Targeted therapies	83	(35.2%)	65	(31.4%)	18	(62.1%)	0.002	99	(47.4%)	78	(43.3%)	21	(72.4%)	0.007	0.012
Targeted therapies group							0.011							0.016	0.050
Not used	153	(64.8%)	142	(68.6%)	11	(37.9%)		110	(52.6%)	102	(56.7%)	8	(27.6%)		
TNF inhibitor	37	(15.7%)	30	(14.5%)	7	(24.1%)		37	(17.7%)	31	(17.2%)	6	(20.7%)		
non-TNF bDMARDs	27	(11.4%)	21	(10.1%)	6	(20.7%)		37	(17.7%)	27	(15.0%)	10	(34.5%)		
JAK inhibitor	19	(8.1%)	14	(6.8%)	5	(17.2%)		25	(12.0%)	20	(11.1%)	5	(17.2%)		

Data were analyzed using the chi-square or Mann–Whitney U test and are presented as number with percentage [n (%)] or median (interquartile range).

SLE, systemic lupus erythematosus; CKD, chronic kidney disease; CAD, coronary artery disease; CVA, cerebrovascular accident; COPD, chronic obstructive pulmonary disease; DM, diabetes mellitus; TNF, tumor necrosis factor; bDMARDs, biologic disease-modifying antirheumatic drugs; JAK, Janus kinase.

### Early immunogenicity in rheumatic patients vaccinated against COVID-19

In order to determine early immunogenicity, we analyzed the anti-SARS-CoV-2 IgG level in AIRD patients after the first dose of COVID-19 vaccination. Compared with those receiving the first dose of AZD1222, participants receiving the first dose of mRNA-1273 had a higher anti-SARS-CoV-2 IgG level (**Table 2**). Compared with non-users, patients using prednisolone-equivalent glucocorticoid dose >5 mg/day, sulfasalazine, and targeted therapies including TNF, non-TNF and JAK inhibitors had a lower anti-SARS-CoV-2 IgG level.

**Table 2:** Multivariate analysis of anti-SARS-CoV-2 IgG level in patients with rheumatic diseases following the first dose of COVID-19 vaccines.

	Univariate analysis				Multivariate analysis			
	β	95% CI		*p* value	β	95% CI		*p* value
Age	-0.646	(-1.25,	-0.037)	0.038	-0.46	(-1.18,	0.25)	0.204
Sex								
Female	Reference							
Male	-16.99	(-36.62,	2.63)	0.089				
Diagnosis								
Healthy controls	Reference				Reference			
SLE	-34.58	(-70.13,	0.96)	0.056	-29.22	(-66.97,	8.53)	0.129
Rheumatoid arthritis	-75.16	(-105.83,	-44.50)	<0.001	-29.22	(-70.30,	11.86)	0.162
Others	-48.86	(-81.71,	-16.01)	0.004	-28.39	(-63.50,	6.72)	0.112
Medications								
Glucocorticoids								
Not used	Reference				Reference			
≤5 mg/day	-32.40	(-66.95,	2.14)	0.066	-19.99	(-53.80,	13.82)	0.245
>5 mg/day	-35.15	(-54.34,	-15.96)	<0.001	-25.83	(-45.58,	-6.08)	0.011
Methotrexate	-42.32	(-60.97,	-23.67)	<0.001	-22.18	(-43.11,	-1.25)	0.038
Leflunomide	-27.93	(-68.82,	12.96)	0.180				
Sulfasalazine	-37.62	(-58.96,	-16.27)	0.001	-19.49	(-41.57,	2.59)	0.083
Hydroxychloroquine	-18.17	(-36.87,	0.53)	0.057				
Azathioprine	14.79	(-18.78,	48.37)	0.386				
Mycophenolate mofetil/acid	-16.79	(-68.49,	34.91)	0.523				
Cyclosporine	-6.01	(-45.65,	33.63)	0.765				
Targeted therapies	-43.70	(-61.74,	-25.67)	<0.001				
Targeted therapies group								
Not used	Reference				Reference			
TNF inhibitor	-38.53	(-62.33,	-14.73)	0.002	-21.01	(-46.52,	4.51)	0.106
Non-TNF bDMARD	-47.08	(-75.77,	-18.40)	0.001	-30.01	(-60.51,	0.50)	0.054
JAK inhibitor	-48.16	(-75.68,	-20.65)	0.001	-20.82	(-51.38,	9.74)	0.181
Vaccine								
ChAdOx1 nCoV-19/AZD1222	Reference				Reference			
mRNA-1273	14.70	(-3.97,	33.38)	0.122	35.25	(14.81,	55.68)	0.001

SLE, systemic lupus erythematosus; TNF, tumor necrosis factor; bDMARDs, biologic disease-modifying antirheumatic drugs; JAK, Janus kinase; β, regression coefficient; 95% CI, 95% confidence intervals.

After adjusting for potential confounders, the serum level of anti-SARS-CoV-2 IgG increased by 35.25 U/mL in patients who received mRNA-1273 than those who received AZD1222. Compared with those without prednisolone use, the anti-SARS-CoV-2 IgG level decreased by 25.83 U/mL on average in patients who used prednisolone >5 mg/day. On average, the anti-SARS-CoV-2 IgG level decreased by 22.18 U/mL in patients treated with MTX compared with those without MTX use.

### Immunogenicity of COVID-19 vaccination in RA patients using bDMARDs and tsDMARDs

In order to evaluate the impact of biological agents on immunogenicity of COVID-19 vaccination, we examined the anti-SARS-CoV-2 IgG level in RA patients using different bDMARDs and tsDMARDs. After the first dose of COVID-19 vaccination, the seropositivity rate of anti-SARS-CoV-2 IgG was 90.6%, 65.4%, 62.5%, and 70.0% (*p*=0.062) (**Figure 1C**), and the average anti-SARS-CoV-2 IgG level was 27.31 (4.04-59.10), 4.92 (0.40-19.68), 1.43 (0.40-9.20), and 1.99 (0.55-7.49) U/ml in RA patients using csDMARDs, anti-TNF-α bDMARDs, non-anti-TNF-α bDMARDs, and JAK inhibitors, respectively. The anti-SARS-CoV-2 IgG level after the first dose of vaccination was markedly lower in RA patients who received non-anti-TNF-α bDMARDs and JAK inhibitor when compared with participants who received csDMARDs (*p*=0.004). After the second dose of COVID-19 vaccination, the seropositivity rate and the anti-SARS-CoV-2 IgG level in RA patients was both uplifted to a large extent, except for the seropositivity rate in RA patients receiving non-anti-TNF-α bDMARDs, compared with those receiving csDMARDs (77.4% vs. 100%, p=0.006).

### Adverse reactions and patient self-reported disease activities after COVID-19 vaccination

Only non-serious adverse reactions occurred in either of the vaccine groups in AIRD patients. There were less fever, chills and fatigue in rheumatic patients receiving mRNA-1273 than those receiving AZD1222 (**Table 3**). Disease activities of rheumatic diseases by self-reported and by physician global assessments both remained stable in most patients who received COVID-19 vaccination, and there was no disparity in rheumatic disease activities after either AZD1222 or mRNA-1273 vaccination (**Table 3** and **Supplementary Table 1**).

**Table 3:** Adverse reactions and rheumatic disease activities in rheumatic patients after COVID-19 vaccines.

	ChAdOx1 nCoV-19/AZD1222		mRNA-1273		*p* value
	(n=236)		(n=209)		
Local adverse reactions					
Pain	83	-35.20%	84	-40.20%	0.32
Erythema	10	-4.20%	8	-3.80%	1
Swelling	43	-18.20%	44	-21.10%	0.527
Itch	17	-7.20%	16	-7.70%	1
Stinging	9	-3.80%	7	-3.30%	0.994
Systemic adverse reactions					
Fever	44	-18.60%	14	-6.70%	**<0.001**
Anorexia	6	-2.50%	5	-2.40%	1
Vomiting	4	-1.70%	5	-2.40%	0.74
Rhinorrhea	0	0.00%	1	-0.50%	0.47
Cough	0	0.00%	2	-1.00%	0.22
Muscle aches	59	-25.00%	41	-19.60%	0.214
Joint pain	35	-14.80%	25	-12.00%	0.456
Chills	33	-14.00%	8	-3.80%	**<0.001**
Fatigue	64	-27.10%	34	-16.30%	**0.008**
Headache	46	-19.50%	27	-12.90%	0.082
Allergy	10	-4.20%	2	-1.00%	0.066
Hypersomnia	22	-9.30%	12	-5.70%	0.215
Self-reported rheumatic disease activities					0.423
Improving	10	-5.30%	5	-2.50%	
Stable	155	-81.60%	173	-86.90%	
Worsening	20	-10.50%	17	-8.50%	
Not sure	5	-2.60%	4	-2.00%	
Physician global assessment					0.516
Stable or improving	167	-87.90%	180	-90.50%	
Worsening	23	-12.10%	19	-9.50%	

Data were analyzed using the chi-square and are presented as number with percentage [n (%)].

### ScRNA-seq of RA patients following COVID-19 vaccination

**Figure 2:**
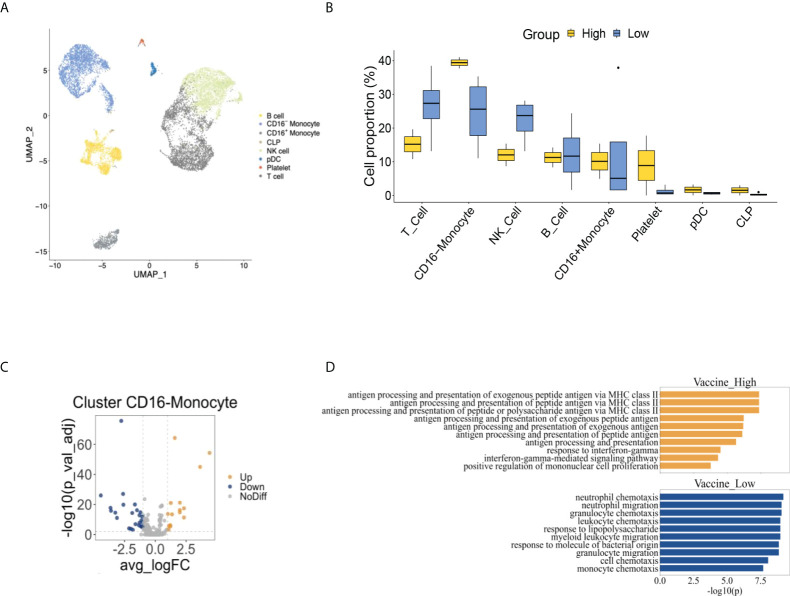
The comprehensive cell atlas of peripheral blood mononuclear cells of rheumatoid arthritis patients with high and low anti-SARS-CoV2-IgG antibodies. **(A)** UMAP visualization of peripheral blood mononuclear cells from rheumatoid arthritis patients. **(B)** The proportion of cell types between high and low anti-SARS-CoV2-IgG antibody groups. **(C)** Volcano plot of CD16-monocyte showed differential expressed genes of high anti-SARS-CoV2-IgG antibody group comparing to low anti-SARS-CoV2-IgG antibody group. **(D)** Pathway analysis between high and low anti-SARS-CoV2-IgG antibody groups.

We performed scRNA-seq to explore the difference of cell composition using two RA patients with high anti-SARS-CoV2-IgG antibodies (one used no biologics, and one used etanercept) and four patients with low antibodies (one used abatacept, one used rituximab, and two used tofacitinib). The detailed clinical information of these patients was listed in **Supplementary Table 2**. To identify individual cells, the expression of major phenotype cell markers was analyzed and was shown in **Supplementary Figure 1**. The frequencies of major identified cell types by scRNA-seq were 25.64% CD16^-^ monocyte, 5.67% CD16^+^ monocyte, 28.59% T cell, 12.73% B cell, 24.46% natural killer (NK) cell, 1.85% platelet, 0.59% plasmacytoid dendritic cell (pDC) and 0.47% common lymphoid progenitor (CLP) (**Figure 2A**). The individual cell distribution of each patient was shown in **Supplementary Figure 2**. The cell proportion of PBMC in RA patients were further confirmed by flow cytometric analysis, which revealed 26.8% T cells, 22.8% CD3^-^CD56^+^NK cells, and 26.1% monocytes (22.8% CD16^-^ monocytes and 3.3% CD16^+^ monocytes) in PBMC from RA patients (**Supplementary Figure 3**). There were more CD16^-^monocytes and less NK cells and T cells in high anti-SARS-CoV2-IgG antibody group than low antibody group by scRNA-seq, though there was no statistical significance (**Figure 2B**).

### Comparisons of the cell composition between healthy controls and RA patients following COVID-19 vaccination by scRNA-seq

**Figure 3:**
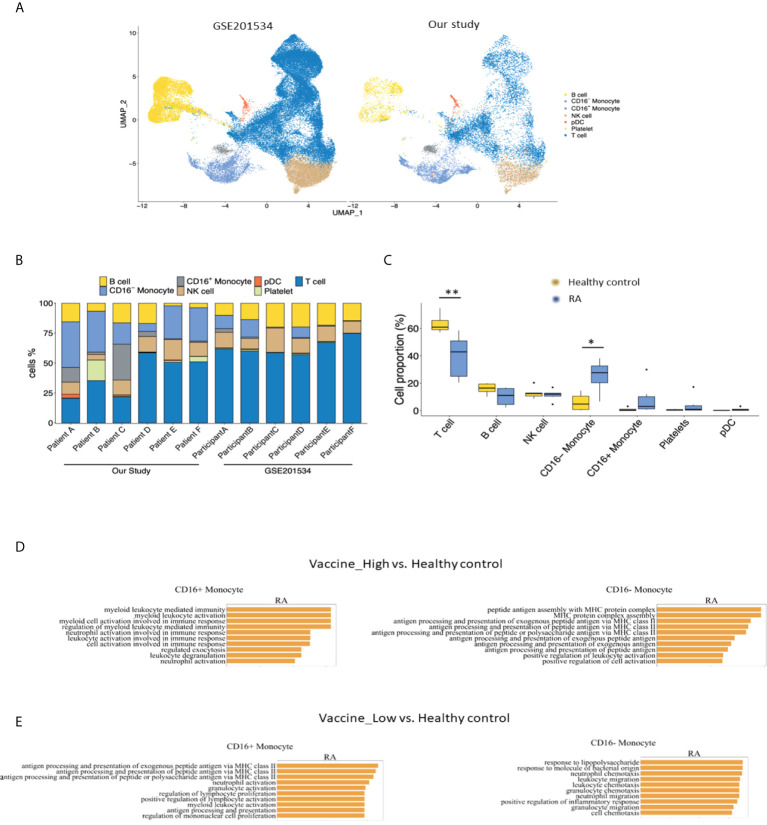
Comparisons of **(A)** cell atlas of peripheral blood mononuclear cells, **(B)** the proportions of cell types from each participant and **(C)** healthy controls from GSE201534 vs. patients with rheumatoid arthritis from our study. Pathway analysis of CD16+ and CD16-monocyte between **(D)** high anti-SARS-CoV2-IgG antibody group and **(E)** low anti-SARS-CoV2-IgG antibody group from our study vs. healthy control from GSE201534. .

To compare the immunogenicity following COVID vaccination between healthy controls and patients with RA, we analyzed scRNA-seq data from public dataset (GSE201534) and our cohort. As depicted in **Figures 3A–C**, cell atlas and the cell proportions among each participant of our study and GSE201534 demonstrated an increased CD16^-^ monocytes but decreased T cell composition between RA patients and heathy controls.

### Enriched pathways between high and low anti-SARS-CoV2-IgG antibody groups in RA patients and healthy controls

We further examined the differentially expressed genes (DEGs) in each major cell population between high and low anti-SARS-CoV2-IgG antibody groups in RA patients and found there were relatively more enriched DEGs in CD16^-^ monocytes than other cells (**Supplementary Figure 4**). We identified 15 up-regulated and 26 down-regulated DEGs between the two groups in CD16^-^monocytes (**Figure 2C**). Through pathway analysis, pathways related to antigen presentation *via* major histocompatibility complex class II (MHC class II) and response to interferon gamma (IFNγ) were enriched in CD16^-^monocytes in high anti-SARS-CoV2-IgG antibody group, while those related to chemotaxis were augmented in low anti-SARS-CoV2-IgG antibody group (**Figure 2D**).

In addition, pathways involved in antigen assembly and processing *via* MHC class II were enriched in CD16^-^ monocytes from high vaccine immunogenicity RA group in comparisons to healthy controls (**Figure 3D**). Similarly, pathways related to chemotaxis were differentially enriched in CD16^-^ monocytes from low vaccine immunogenicity RA patients as compared to healthy controls (**Figure 3E**).

Furthermore, we also performed analysis on B cells since B cells were the main cell type for antibodies production. However, there were no significant cell proportion difference in B cell sub-groups between high and low anti-SARS-CoV-2 IgG groups, and no DEGs could be found between the two groups, either (**Supplementary Figure 5**).

### Different cell-cell interaction between high and low anti-SARS-CoV2-IgG antibody groups in RA patients

**Figure 4:**
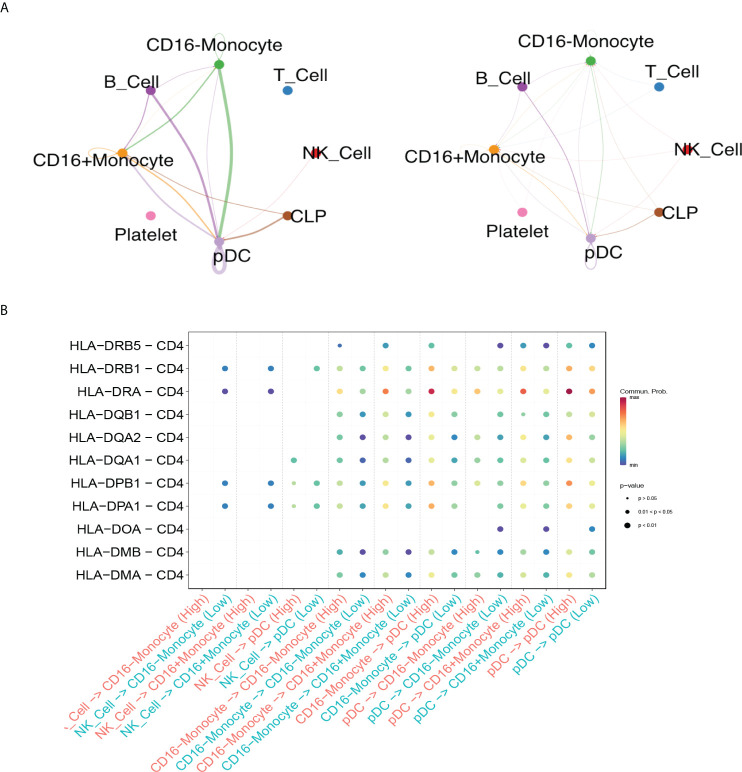
Differential crosstalks between high and low anti-SARS-CoV2-IgG antibody groups among each cell populations in rheumatoid arthritis patients. **(A)** Circle plot shows the MHC class II pathway outgoing and incoming signaling among high anti-SARS-CoV2-IgG antibody group (left) and low anti-SARS-CoV2-IgG antibody group (right); **(B)** Bubble plot shows the selected ligand-receptor interactions between NK cells, monocytes and pDC.

Through cell-cell interaction analysis, we found nineteen interactions enriched significantly in AIRD patients with high anti-SARS-CoV-2 IgG level, while nine interactions enriched in those with low anti-SARS-CoV-2 IgG level (**Supplementary Figure 6**). There were stronger interactions *via* MHC class II pathway between CD16^-^monocytes, CD16^+^monocytes, B cells and pDCs in high anti-SARS-CoV2-IgG antibody group than in low anti-SARS-CoV2-IgG antibody group (**Figure 4**). HLA-DRA and CD4 interaction was vigorous among all identified MHC class II pathway and was more enhanced in high anti-SARS-CoV2-IgG antibody group than in low antibody group (**Figure 4B**). The other crosstalks between high and low anti-SARS-CoV2-IgG antibody groups among each cell populations in RA patients *via* IFNγ and CCL pathway, and the associated ligand-receptor interactions were shown in **Supplementary Figure 7**.

## Discussion

Our study demonstrated an anti-SARS-CoV-2 IgG seropositivity rate of 85.34% and 98.20% in AIRD patients and in healthy controls, respectively. The serum anti-SARS-CoV-2 IgG level after the first dose of the vaccine was higher in participants who received mRNA-1273 than in those who received AZD1222 vaccine; however, the IgG level was lower in patients who received a prednisolone-equivalent dose >5 mg/day or MTX than in non-users. In RA patients, a markedly lower anti-SARS-CoV-2 IgG level was observed in participants who were treated with non-anti-TNF-α bDMARDs and JAK inhibitors. Higher proportion of CD16^-^ monocytes were observed in RA patients following COVID-19 vaccination in comparison to healthy controls. Pathways related to antigen presentation *via* MHC class II in CD16^-^ monocytes, especially HLA-DRA and CD4 interaction, might be associated with higher anti-SARS-CoV2-IgG level in RA patients.

In the present study, we found that anti-SARS-CoV-2 IgG levels were remarkably higher after the first dose in rheumatic disease patients who received mRNA-1273 vaccines than in those who received AZD1222. Prendecki et al. demonstrated that, in patients with rheumatic disease, the anti-SARS-CoV-2 IgG levels of those who received BNT2b162 were numerically higher than those of patients who received the vector vaccine AZD1222 (22). Other reports have revealed similar information among the general population. One study in Germany showed that, between weeks 6 and 15, the anti-spike IgG level was higher after the first vaccination with the mRNA-based vaccines mRNA-1273 or BNT162b2, than with the vector vaccine AZD1222 (23). Among individuals who received AZD1222 as their first vaccine, a strong increase in the anti-spike IgG level was observed in those who received a subsequent dose of mRNA-based vaccine than a vector vaccine. However, the correlation between vaccine types and the anti-SARS-CoV-2 IgG level remains controversial, and there is a paucity of data in immunocompromised populations. Furthermore, differences in the neutralization capacities, along with the clinical influence of anti-spike IgG, between different vaccine types are unclear. Further research based on the impact of different vaccine types in patients with rheumatic disease are warranted to provide information for policymakers with regard to vaccine allocation in high-risk populations.

Our results demonstrated that, compared with non-users, the anti-SARS-CoV-2 IgG level was lower in AIRD patients receiving a prednisolone-equivalent glucocorticoid dose >5 mg/day or MTX. The initial analysis of the COVaRiPAD study revealed fewer circulating plasmablasts and a 10-fold reduction of antibody response following mRNA-based vaccination in patients with rheumatic disease receiving glucocorticoids (24). Furthermore, the seropositivity rate decreased to 65% in patients treated with MTX but returned to 92% after discontinuation of prednisolone. Similar findings have been reported from SLE patients following influenza vaccination when a prednisolone dose >20 mg/day was administered (25). We proposed that, in patients with stable underlying rheumatic disease, a pre-vaccination reduction in the glucocorticoid dose could elicit adequate immunogenicity. The dose-dependent effect of glucocorticoids on immunogenicity following COVID-19 vaccination warrants further investigation. Furthermore, Haberman et al. reported that, in a New York cohort, the seropositivity rate in patients with rheumatic disease who were treated with MTX was remarkably lower than that of patients without MTX use (72.0% vs. 92.3%, *p*=0.023), and the anti-SARS-CoV-2 IgG level tended to be lower in patients with rheumatic disease who were treated with MTX (9). Additionally, MTX use was associated with attenuated CD8+ T-cell activation, which plays a protective role against SARS-CoV-2 infection in a study of rhesus macaques (26). Therefore, impaired antibody production as well as attenuation of CD8+ T-cell response by MTX diminished the immunogenicity of COVID-19 vaccines in patients with rheumatic disease. Nonetheless, it remains unclear whether the temporary discontinuation of MTX could improve the immunogenicity of COVID-19 vaccines, although this possibility is conceivable because higher antibody levels were achieved in RA patients after MTX discontinuation for 2 weeks for influenza vaccination (27). Accordingly, additional vaccine boosters could be considered in patients treated with glucocorticoids or MTX to achieve adequate immunization against SARS-CoV-2.

A notably lower level of anti-SARS-CoV-2 IgG was observed in RA patients treated with JAK inhibitors. However, only a few studies have investigated the impact of JAK inhibitors on the efficacy of COVID-19 vaccination with contradictory results. Deepak et al. suggested that the antibody titers might be reduced 6.6-fold in patients using JAK inhibitors (24). JAK inhibitors target small molecules and are associated with alteration of interferon signaling, change in T- and B-cell counts, and reduction of natural killer cell counts, all of which might lead to decreased immunogenicity after vaccination (25, 28). Nonetheless, Furer et al. reported that there was no difference in the seropositivity rate after BNT162b2 vaccination between rheumatic patients using JAK inhibitors and healthy controls (8). In studies investigating the influenza and pneumococcal vaccine response, immunogenicity was unaltered by JAK inhibitor treatment (29, 30). Further studies are needed to explore the clinical impact of JAK inhibitors on the immunogenicity of COVID-19 vaccination.

Our study revealed that pathways related to antigen presentation *via* MHC class II in CD16^-^monocytes, especially HLA-DRA and CD4 interaction, and pathways response to IFNγ might be associated with higher anti-SARS-CoV2-IgG level in RA patients, while pathways related to chemotaxis were kept in those with low anti-SARS-CoV2-IgG antibody. Sureshchandra S et al. demonstrated increasing spike-specific B cells, antigen-specific CD4^+^ T cells, and CD8^+^ T cells by scRNA-seq analysis in healthy subjects after mRNA vaccines and those after COVID-19 infection (31). Kramer KJ and the colleagues focused on adaptive immunity and indicated enlarged population of antigen-specific memory CD4+ and CD8+ T cells, and IgA and IgG memory B cells specific to SARS-CoV-2 in healthy participants after BNT162b2 mRNA vaccines (32). Those studies highlighted the importance of antigen presentation and antibodies productions following mRNA vaccines in healthy individuals. In RA patients after COVID-19 vaccination, we found CD16^-^ monocytes might be one of the key players in antigen presentation. Generally, CD16^-^monocytes were classical monocytes and were considered to be primed for chemotaxis and phagocytosis, while intermediate monocytes were responsible for antigen presentation (33, 34). Nevertheless, our results and analysis of public datasets (GSE201534) might imply that more CD16^-^monocytes in RA patients with high anti-SARS-CoV-2 IgG level turned to perform antigen presentation *via* HLA-DRA of MHC class II rather than chemotaxis. Lee J et al. demonstrated that although the intermediate monocytes exhibited the most MHC molecules, classical monocytes might be recruited as antigen presentation cells and express MHC class II to a great extent after stimulation of inflammatory cytokines, such as IFNγ, granulocyte-macrophage colony-stimulating factor (GM-CSF) and IL-4 (35). Since HLA-DRA was one of the MHC class II alpha subunits, which might interact with CD4+ cells and further promote the antibodies production by B cells. Lower level of anti-SARS-CoV-2 IgG and less enriched pathways related antigen presentation in CD6-monocytes were detected in RA patients using non-anti-TNF-α bDMARDs and JAK inhibitors. JAK inhibitors could repress JAK-STAT pathway mediated by GM-CSF in innate immune cells including monocytes. Multiple inflammatory cytokines such as GM-CSF and interferon were direct targets by JAK inhibitors and may possibly suppress the promotion of antigen presentation function in CD16^-^monocytes (36).

This study has some limitations. First, our study was limited in the sample size, and a selection bias might exist. We are still expanding the case numbers and more studies are underway. Second, most participants in our study received the first dose of COVID-19 vaccine but not the second dose due to a shortage of vaccine supply in Taiwan during the initial period of study enrollment. Third, the neutralizing capacity and T-cell response were not evaluated in our study. As a humoral immune response does not necessarily represent the complete picture of anti-COVID immunity and the efficacy of COVID-19 vaccines, further research is needed to explore the issue.

## Conclusion

After the first dose COVID-19 vaccination, the anti-SARS-CoV-2 IgG level in patients with rheumatic diseases receiving mRNA-1273 was higher than that with AZD1222, and mRNA-based vaccines could be prioritized for AIRD patients to elicit adequate humoral immunity against SARS-CoV-2. A prednisolone-equivalent glucocorticoid dose >5 mg/day, MTX use, and RA patients treated with non-anti-TNF-α bDMARDs or JAK inhibitors were risk factors for lower immunogenicity. Moreover, enriched pathways related to antigen presentation *via* MHC class II in CD16^-^monocytes might be associated with higher anti-SARS-CoV2-IgG level in RA patients.

## Data availability statement

The datasets presented in this study can be found in online repositories. The name of the repository and accession number can be found below: NCBI Gene Expression Omnibus, accession number GSE203081.

## Ethics statement

This study was conducted in accordance with the Declaration of Helsinki and was approved (CF21297B) by the Institutional Review Board of Taichung Veterans General Hospital, Taiwan. The patients/participants provided their written informed consent to participate in this study.

## Author contributions

Y-MC, T-HH, and Y-JC conceived and designed the study. Y-MC and Y-JC performed the literature search, interpretation of data and drafted the manuscript. P-LC, H-WC, and J-PC conducted data extraction, methodological quality assessments and performed the analysis. P-LC performed the single-cell RNA sequencing experiments. Y-HC, W-NH, H-HC, C-TL, K-TT, W-TH, and T-YH performed critical revision of the manuscript for important intellectual content. All authors read and approved the final version of submitted manuscript.

## Funding

This study was financially supported by grants from Taichung Veterans General Hospital (TCVGH-1107302D) and Ministry of Science and Technology (111-2314-B-075A-003 -MY3), Taiwan.

## Acknowledgments

The authors thank the Biostatistics Task Force of Taichung Veterans General Hospital for their assistance with the statistical analysis in this study.

## Conflict of interest

The authors declare that the research was conducted in the absence of any commercial or financial relationships that could be construed as a potential conflict of interest.

## Publisher’s note

All claims expressed in this article are solely those of the authors and do not necessarily represent those of their affiliated organizations, or those of the publisher, the editors and the reviewers. Any product that may be evaluated in this article, or claim that may be made by its manufacturer, is not guaranteed or endorsed by the publisher.
