# Supplementary material for: Single-cell RNA sequencing to decipher the immunogenicity of ChAdOx1 nCoV-19/AZD1222 and mRNA-1273 vaccines in patients with autoimmune rheumatic diseases
Source: Front Immunol. 2022 Aug 1;13:920865. doi: 10.3389/fimmu.2022.920865 (PMC9376226; doi:10.3389/fimmu.2022.920865)
Supplement: Supplementary file 1 [file DataSheet_1.pdf]

Supplementary Figure 1. The expression of major phenotype cell markers in UMAP space.

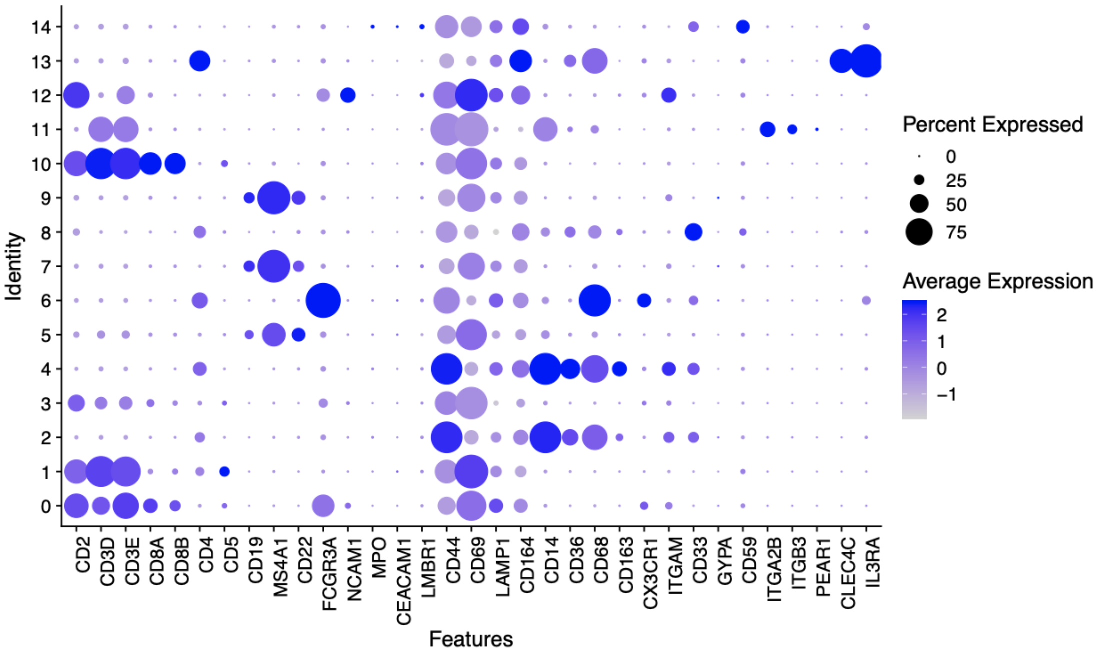

Supplementary Figure 2. The individual cell distribution of the six RA patients underwent single-cell RNA sequencing.

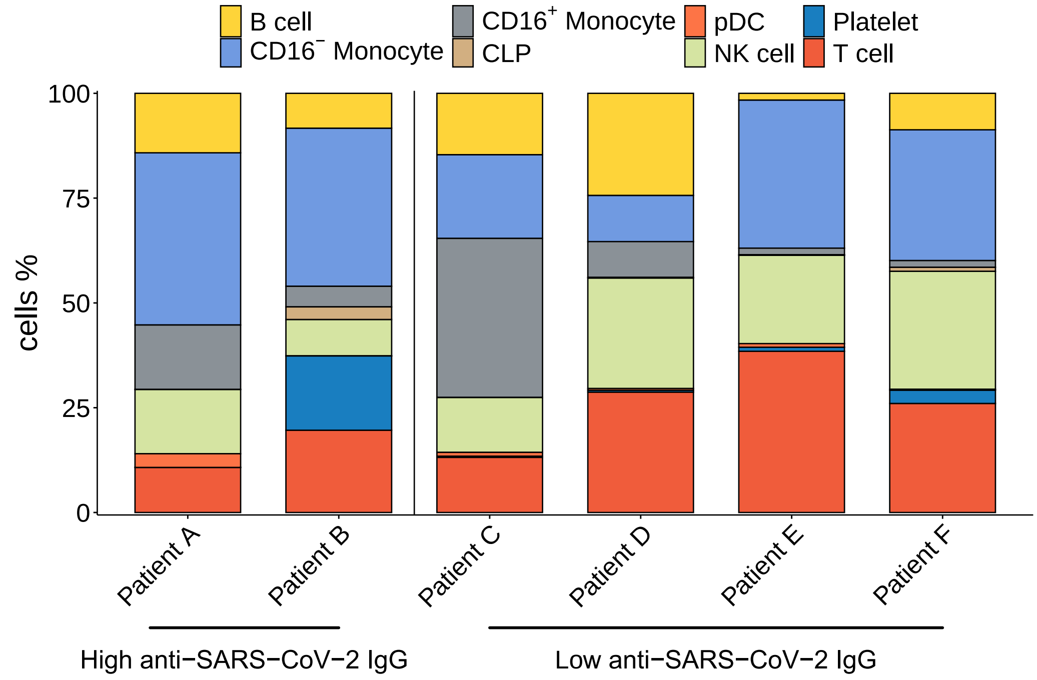

Supplementary Figure 3. Representative flow cytometric analysis of PBMC in RA patients. (A) Intact cells were identified by forward scatter (FSC-A) and side scatter (SSC-A) characteristics. P3 and P4 cell population were used for further analysis of T cells, NK cells, and monocytes, respectively. The cell proportion of PBMC in RA patients were (B) 26.8% T cells, (C) 22.8% $CD3^{-}CD56^{+}$NK cells, and (D) 26.1% monocytes, (E) of which 22.8% were $CD16^{-}$ monocytes and 3.3% were $CD16^{+}$ monocytes.

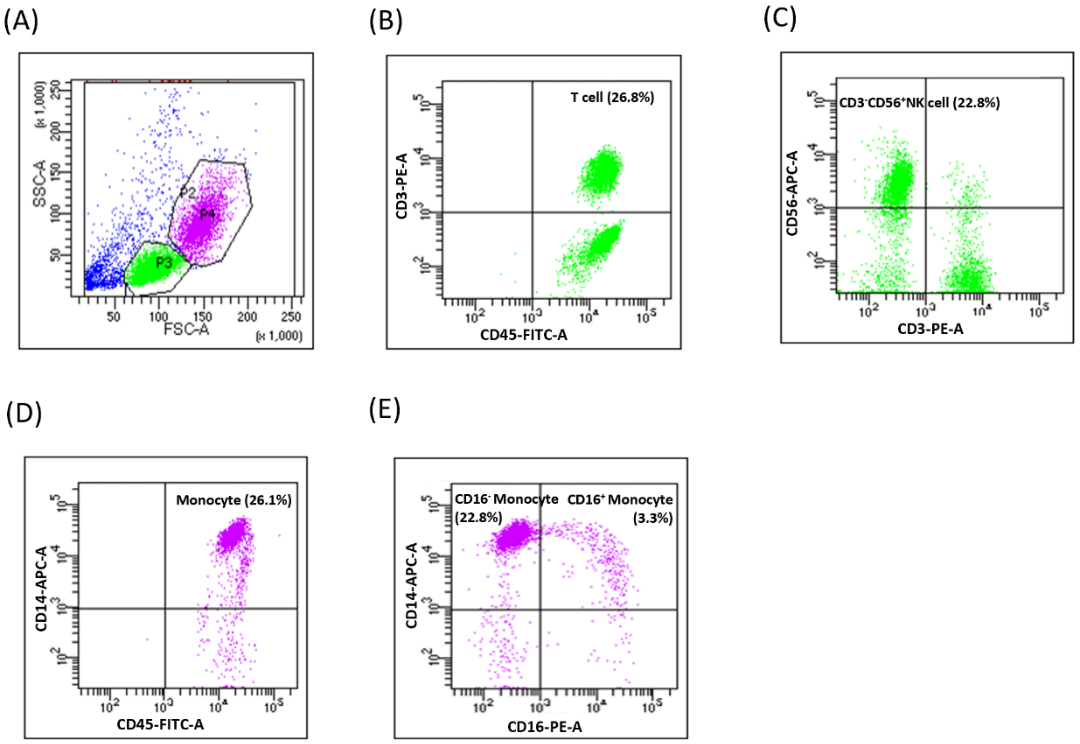

Supplementary Figure 4. The differentially expressed genes (DEGs) in each major cell population between high and low anti-SARS-CoV2-IgG antibody groups in patients with rheumatoid arthritis.

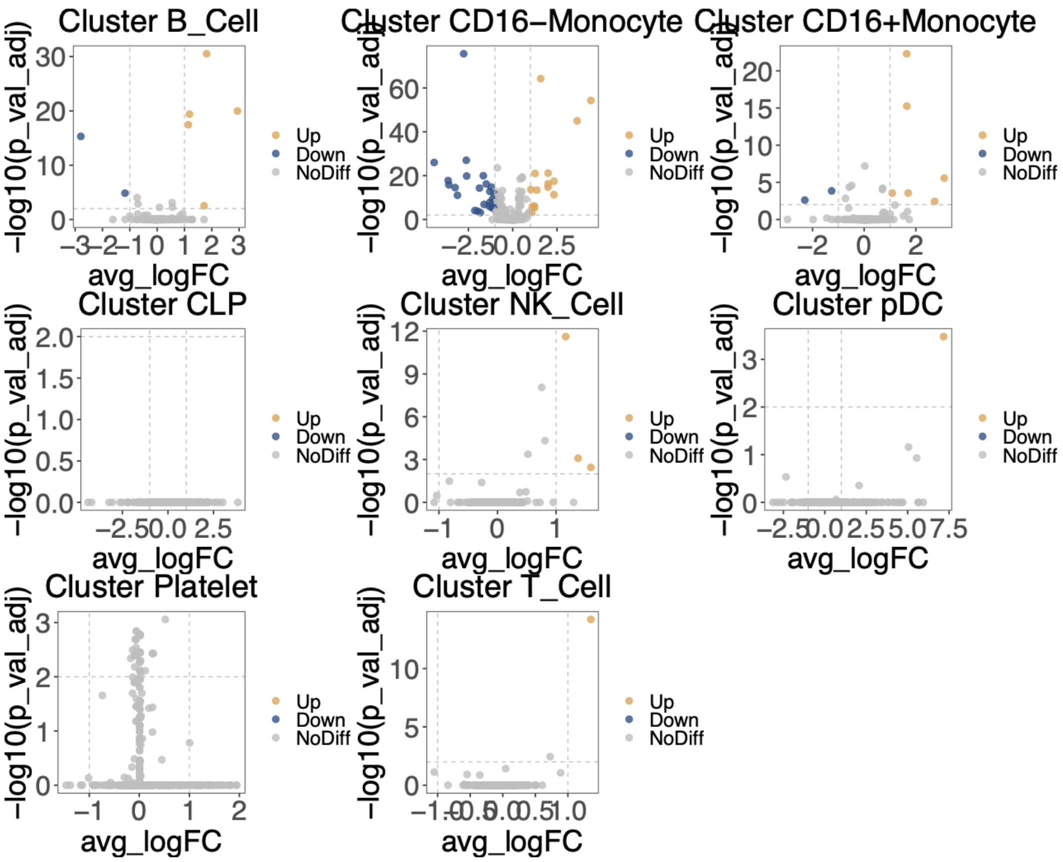

Supplementary Figure 5. The cell atlas and DEGs of B cells in RA patients with high and low anti-SARS-CoV2-IgG antibodies.

A) UMAP visualization of B cells from RA patients. B) The individual B cell subcluster distribution of the six RA patients underwent single-cell RNA sequencing. C) Volcano plot showed the DEGs in B cells between high and low anti-SARS-CoV2-IgG antibody groups in RA patients. Abbreviations: RA: rheumatoid arthritis, DEGs: differentially expressed genes, DN: double negative B cells.

(A)

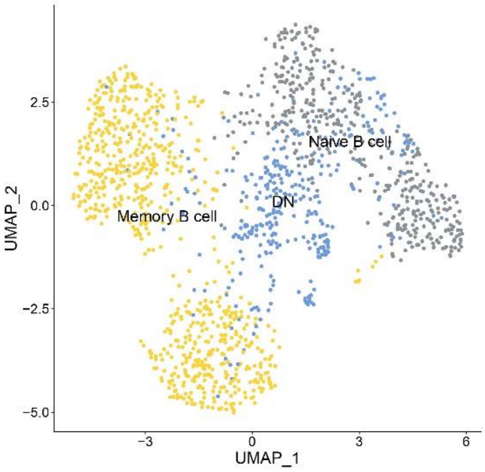

(B)

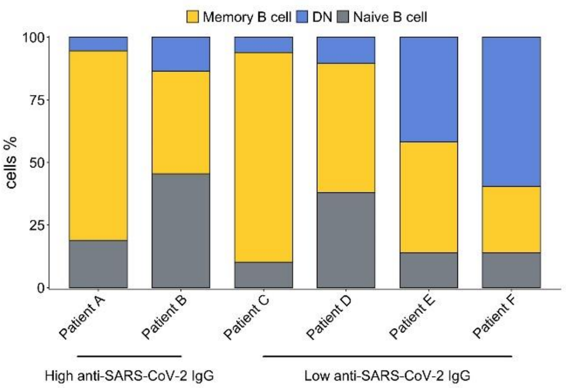

(C)

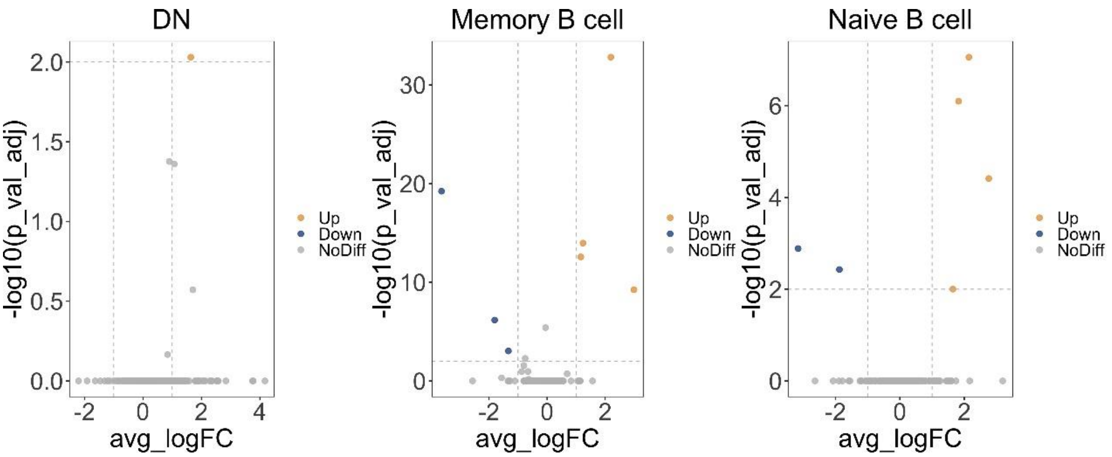

Supplementary Figure 6. The significant cell-cell interactions with differential enrichment between RA patients with high and low anti-SARS-CoV-2 IgG levels.

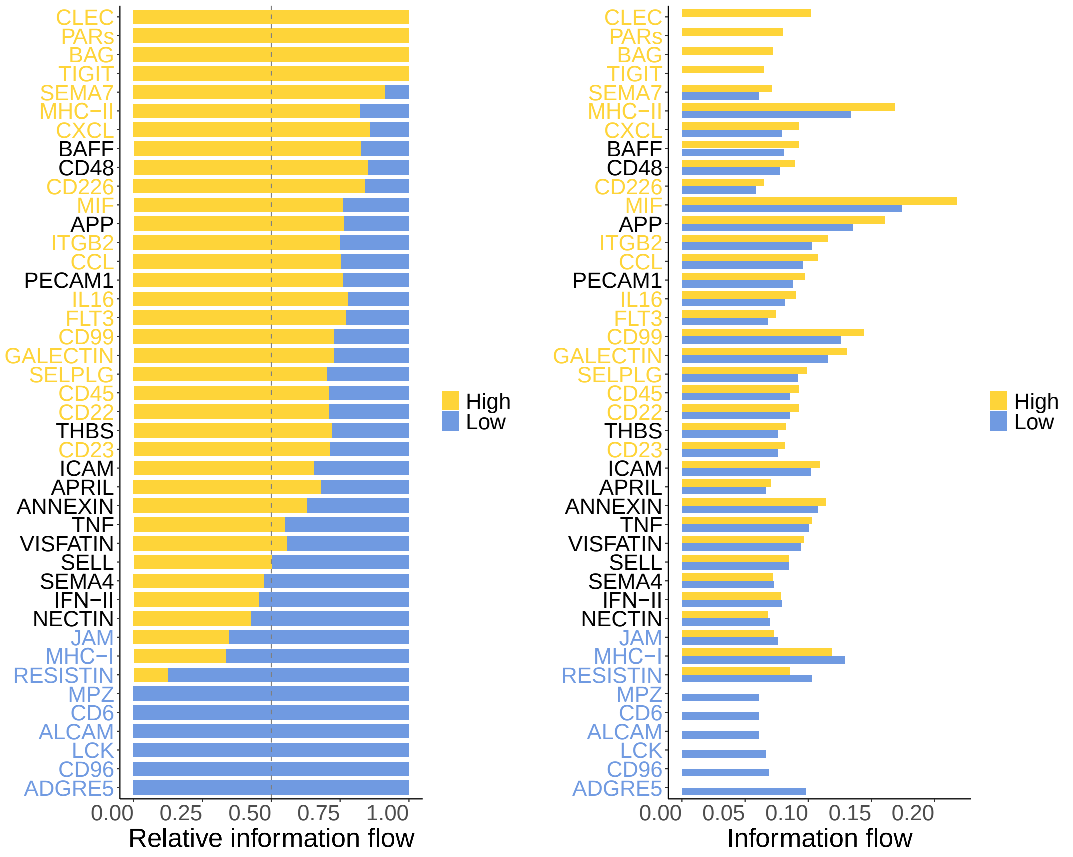

Supplementary Figure 7. Different crosstalks between high and low anti-SARS-CoV2-IgG antibody groups among each cell populations in rheumatoid arthritis patients. (A) Circle plot shows the IFNγ pathway outgoing and incoming signaling among high anti-SARS-CoV2-IgG antibody group (upper) and low anti-SARS-CoV2-IgG antibody group (lower); (B) Circle plot shows the CCL pathway outgoing and incoming signaling among high anti-SARS-CoV2-IgG antibody group (upper) and low anti-SARS-CoV2-IgG antibody group (lower); (C) Bubble plot shows the selected ligand-receptor interactions.

(A)

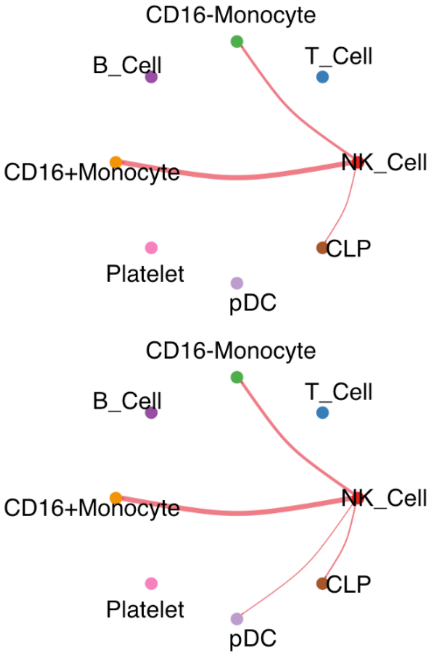

(B)

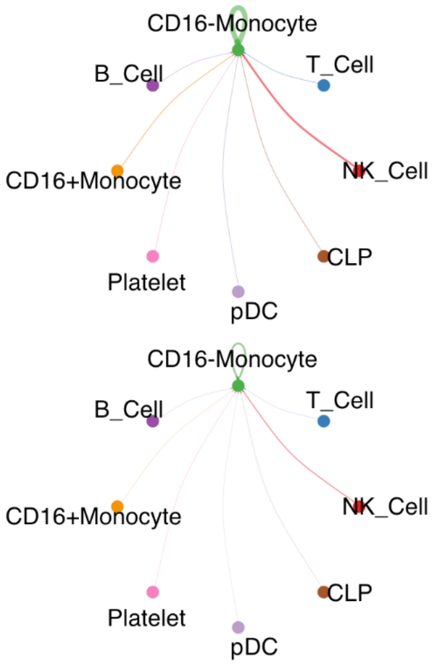

(C)

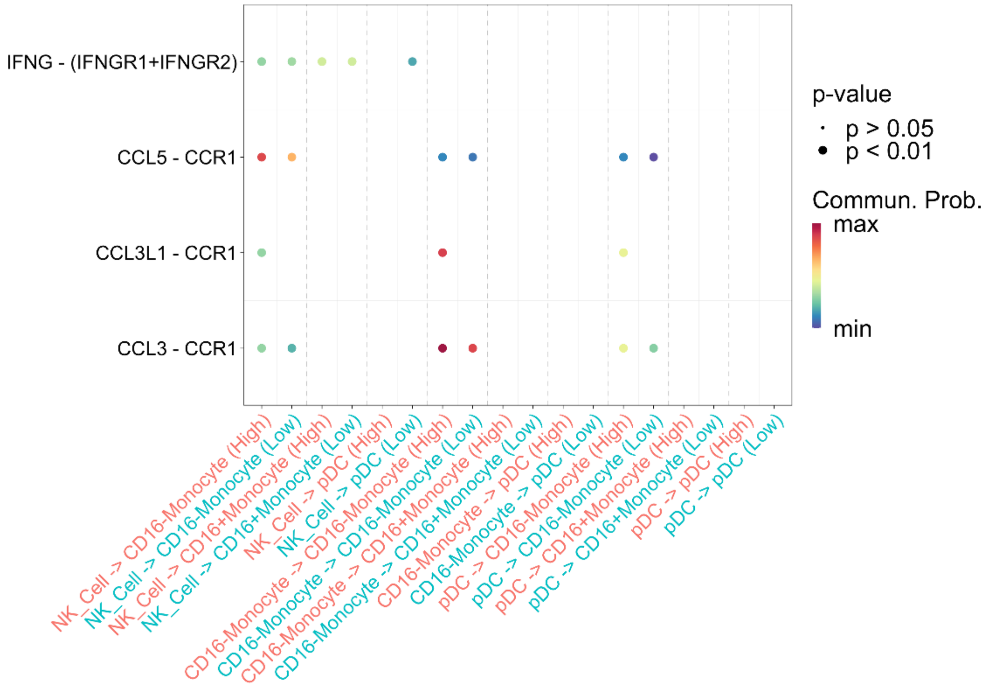

Supplementary Table 1. The disease activities of rheumatic patients among different rheumatic diseases after COVID-19 vaccinations.

	All rheumatic patients			Systemic lupus erythematosus			Rheumatoid arthritis			Sjogren's syndrome			Psoriasis and psoriatic arthritis			Ankylosing spondylitis			Others		
	AZD1222 (n=190)	mRNA-1273 (n=199)	*p* value	AZD1222 (n=39)	mRNA-1273 (n=43)	*p* value	AZD1222 (n=82)	mRNA-1273 (n=112)	*p* value	AZD1222 (n=12)	mRNA-1273 (n=17)	*p* value	AZD1222 (n=17)	mRNA-1273 (n=6)	*p* value	AZD1222 (n=19)	mRNA-1273 (n=11)	*p* value	AZD1222 (n=21)	mRNA-1273 (n=10)	*p* value
**Self-reported**			0.423			0.570			0.229			0.767			0.679			0.445			0.664
Improving	10 (5.3%)	5 (2.5%)		0 (0.0%)	1 (2.3%)		6 (7.3%)	3 (2.7%)		1 (8.3%)	1 (5.9%)		1 (5.9%)	0 (0.0%)		1 (5.3%)	0 (0.0%)		1 (4.8%)	0 (0.0%)	
Stable	155 (81.6%)	173 (86.9%)		36 (92.3%)	36 (83.7%)		63 (76.8%)	95 (84.8%)		8 (66.7%)	14 (82.4%)		15 (88.2%)	6 (100.0%)		15 (78.9%)	11 (100.0%)		18 (85.7%)	10 (100.0%)	
Worsening	20 (10.5%)	17 (8.5%)		2 (5.1%)	3 (7.0%)		13 (15.9%)	14 (12.5%)		2 (16.7%)	1 (5.9%)		1 (5.9%)	0 (0.0%)		2 (10.5%)	0 (0.0%)		1 (4.8%)	0 (0.0%)	
Not sure	5 (2.6%)	4 (2.0%)		1 (2.6%)	3 (7.0%)		0 (100.0%)	0 (100.0%)		1 (8.3%)	1 (5.9%)		0 (0.0%)	0 (0.0%)		1 (5.3%)	0 (0.0%)		1 (4.8%)	0 (0.0%)	
**PGA**			0.516			0.342			0.971			0.553			0.462			0.279			1.000
Stable / improving	167 (87.9%)	180 (90.5%)		36 (92.3%)	42 (97.7%)		70 (85.4%)	97 (86.6%)		10 (83.3%)	16 (94.1%)		16 (94.1%)	5 (83.3%)		16 (84.2%)	11 (100.0%)		19 (90.5%)	9 (90.0%)	
Worsening	23 (12.1%)	19 (9.5%)		3 (7.7%)	1 (2.3%)		12 (14.6%)	15 (13.4%)		2 (16.7%)	1 (5.9%)		1 (5.9%)	1 (16.7%)		3 (15.8%)	0 (0.0%)		2 (9.5%)	1 (10.0%)	

Abbreviation: PGA: physician global assessment

Supplementary Table 2. Clinical information of six RA patients underwent single-cell RNA sequencing.

Participants	Age	Gender	Comorbidities	Medications			Types of COVID-19 vaccination	Anti-SARS-CoV-2 IgG level (U/ml)	DAS28-ESR	
				Glucocorticoids	DMARDs	Targeted therapies			before vaccination	after vaccination
Patient A	32	male	—	5 mg/day	—	Etanercept	mRNA-1273	>250	2.39	1.36
Patient B	64	male	—	-	—		AZD1222	>250	2.51	3.39
Patient C	66	female	Hypertension, hyperlipidemia, CKD	5 mg/day	MTX	Tofacitinib	mRNA-1273	1.79	4.61	4.45
Patient D	63	female	—	—	MTX, HCQ	Abatacept	AZD1222	<0.4	2.61	4.05
Patient E	56	male	Hypertension	10 mg/day	—	Rituximab	AZD1222	<0.4	1.98	1.98
Patient F	63	female	—	5 mg/day	HCQ	Tofacitinib	AZD1222	5.95	5.36	5.03

Abbreviations: CKD: chronic kidney disease, DMARDs: disease-modifying antirheumatic drugs, MTX: methotrexate, HCQ: hydroxychloroquine, DAS28-ESR: disease activity score-28 for rheumatoid arthritis with ESR.
